# Enhanced pharmacological efficacy of sumatriptan due to modification of its physicochemical properties by inclusion in selected cyclodextrins

**DOI:** 10.1038/s41598-018-34554-w

**Published:** 2018-11-01

**Authors:** Magdalena Paczkowska, Mikołaj Mizera, Kinga Sałat, Anna Furgała, Piotr Popik, Justyna Knapik-Kowalczuk, Anna Krause, Daria Szymanowska-Powałowska, Zbigniew Fojud, Maciej Kozak, Marian Paluch, Judyta Cielecka-Piontek

**Affiliations:** 1 Department of Pharmacognosy, Poznań University of Medical Sciences, Święcickiego 4, 60–781 Poznań, Poland; 2 Department of Pharmacodynamics, Jagiellonian University Medical College, Medyczna 9, 30–688 Kraków, Poland; 3 Faculty of Health Sciences, Jagiellonian University Medical College, Michałowskiego 12, 31–126 Kraków, Poland; 4 Institute of Physics, University of Silesia, Uniwersytecka 4, 40–007 Katowice, Poland; 5 Pozlab sp. z o.o., Parkowa 2, 60–775 Poznań, Poland; 6 Department of Biotechnology and Food Microbiology, Poznań University of Life Sciences, Wojska Polskiego 48, 60–627 Poznań, Poland; 7 Department of Macromolecular Physics, Adam Mickiewicz University in Poznań, Umultowska 85, 61–614 Poznań, Poland

## Abstract

The study focused on the pharmacological action of sumatriptan, in particular its antiallodynic and antihyperalgesic properties, as an effect of cyclodextrinic inclusion of sumatriptan, resulting in changes of its physicochemical qualities such as dissolution and permeability through artificial biological membranes, which had previously been examined *in vitro* in a gastro-intestinal model. The inclusion of sumatriptan into β-cyclodextrin and 2-hydroxylpropylo-β-cyclodextrin by kneading was confirmed with the use of spectral (fourier-transform infrared spectroscopy (FT-IR); solid state nuclear magnetic resonance spectroscopy with magic angle spinning condition, ^1^H and ^13^C MAS NMR) and thermal (differential scanning calorimetry (DSC)) methods. A precise indication of the domains of sumatriptan responsible for its interaction with cyclodextrin cavities was possible due to a theoretical approach to the analysis of experimental spectra. A high-performance liquid chromatography with a diode-array detector method (HPLC-DAD) was employed to determine changes in the concentration of sumatriptan during dissolution and permeability experiments. The inclusion of sumatriptan in complex with cyclodextrins was found to significantly modify its dissolution profiles by increasing the concentration of sumatriptan in complexed form in an acceptor solution compared to in its free form. Following complexation, sumatriptan manifested an enhanced ability to permeate through artificial biological membranes in a gastro-intestinal model for both cyclodextrins at all pH values. As a consequence of the greater permeability of sumatriptan and its increased dissolution from the complexes, an improved pharmacological response was observed when cyclodextrin complexes were applied.

## Introduction

Migraine is one of the most frequent neurological disorders worldwide. The high prevalence of this disabling disease in the general population generates a strong medical imperative for the search for novel treatment options for migraine attacks and its prevention^1^. Current migraine therapies are mainly non-specific and are characterized by low patient compliance. The only specific anti-migraine drugs, i.e. triptans, effectively relieve migraine pain but their use is often limited by adverse effects, time-and frequency-restricted application and the risk of developing medication overuse headache^2^. In recent years, novel drug targets for migraine have been discovered, e.g. 5-HT_1F_ receptors and calcitonin-gene-related peptide (CGRP) receptors; however, despite this, the superiority of sumatriptan over placebos in the attenuation of headache, nausea, photophobia, and phonophobia in migraineurs is still not well-established. Although of the triptans sumatriptan demonstrates the strongest antiemetic action, an advantage in the treatment of migraine with vomiting^3^, it has the lowest bioavailability, which is a limitation to the achievement of a greater pharmacological effect^4^. Another problem is the bitter taste of sumatriptan that has not been eliminated by the conversion of the compound to a succinate ester, a form found in commercially available pharmaceutical preparations^5^. However, the increased lipophilicity of sumatriptan aimed at an enhancement of its bioavailability negatively affects its physicochemical properties, such as the required level of solubility, important for the development of pharmaceutical formulations.

The pure form of sumatriptan (its base) is a hydrophilic form. After oral administration, this has limited absorption and its ability to penetrate biological barriers is insufficient. Similarly, commercially available the sumatriptan succinate is not fully absorbed from the gastrointestinal tract, as it is extensively metabolized. Thus, sumatriptan succinate also has low bioavailability (about 15%) and a short half-time in the human organism^6^. The aforementioned drawbacks of these two sumatriptan forms do not affect the widespread use of this drug by migraine sufferers. Importantly, the significant pharmacological activity of this drug is the premise for the search for new triptans and new triptan delivery systems. Taking into consideration the above limitations to the use of sumatriptan, cyclodextrins can be viewed as attractive excipients constituting drug delivery systems able to mask the taste, increase solubility, and improve bioavailability in oral, nasal and transdermal administration as well as allowing for gradual dissolution from the inclusion system ensuring slow drug release^7–13^. There have been reports on the application of some cyclodextrins to obtain intranasal compositions^14^. All intranasal complexes of sumatriptan and cyclodextrins (unsubstituted and substituted) demonstrated improved pharmacokinetic parameters (t_max_) after nasal administration as a result of greater solubility and bioavailability following the use of complexes. Similar studies on the possibility of applying cyclodextrins as compounds improving physicochemical properties important for the oral administration of sumatriptan have not, to the best of our knowledge, been published. Reports are available on the development of sumatriptan succinate transdermal delivery systems^8–12^. For example, Balaguer-Fernander obtained transdermal delivery systems of sumatriptan succinate by binding it with methyl cellulose, polyvinyl pyrrolidone, and polyvinyl pyrrolidone-polyvinyl alcohol in the presence of a required adhesive agent that showed activity when administered on pig ear skin^10^. Another method of increasing the solubility of sumatriptan succinate is by obtaining its amorphous form, for which a higher solubility rate has been demonstrated as compared to the crystalline form found in therapeutic applications^15^. Regarding the advantages of using cyclodextrins to develop oral systems of sumatriptan, the present study required an animal model permitting evaluation of the therapeutic effect of the proposed system against the free form of sumatriptan. In order to evaluate the possible benefits of preparing a system of cyclodextrin and the acidic form of sumatriptan, the pharmacological action of the proposed system was also examined in relation to the salt form, sumatriptan succinate, currently used in oral formulations of sumatriptan.

Serotonin 5-HT_1B/D_ receptors have been found on the trigeminal nerve and cranial vessels. Their agonists–triptans–are effective in migraine attack treatment by acting within the trigeminovascular system to restore normal serum levels of the calcitonin gene-related peptide (CGRP)^16^. Since triptans induce vasoconstriction and can attenuate neurogenic inflammation by decreasing the release of substance P and CGRP^17^, in the *in vivo* part of this study we additionally assessed antinociceptive properties of these four forms of sumatriptan in a mouse model of neurogenic pain induced by capsaicin. Capsaicin, an active component of chili peppers, is a potent TRPV1 channel agonist^18^ and TRPV1 antagonists have been identified as potential therapeutics for migraine^19^. Capsaicin injections stimulate the trigeminovascular system and increase c-fos expression in the trigeminal ganglion in a rodent model of migraine^19^. Apart from its influence on TRPV1, it can regulate CGRP expression in the trigeminal ganglion^20^ and CGRP is strongly involved in capsaicin-induced vasodilation^21^, being regarded as one of the major neuropeptides released from activated trigeminal sensory afferents in headache and facial pain disorders^22^. In migraineurs, CGRP facilitates nociceptive transmission in the activated trigeminovascular system, possibly via presynaptic mechanisms^23^ due to the release of various neurotransmitters, neuropeptides and vasodilators that induce severe pain responses.

The aim of the present study was to prepare and characterize a sumatriptan (SUM) inclusion complex with β-cyclodextrin (SUM-BCD) and 2-hydroxylpropylo-β-cyclodextrin (SUM-HPBCD) in terms of changes resulting from the formation of the complex in the dissolution profiles and permeability of sumatriptan through biological membranes in the gastrointestinal tract. In the *in vivo* part of the present study, the antiallodynic and antihyperalgesic properties and related pleiotropic actions of four forms of sumatriptan such as sumatriptan (SUM), sumatriptan succinate (SUM-SUCC) and sumatriptan complexes with cyclodextrins (SUM-BCD, SUM-HPBCD) were compared after oral administration in a mouse model of migraine pain.

## Results

There are three areas of interest regarding the results of the present study: (1) the procedure of identifying SUM-BCD and SUM-HPBCD inclusion complexes; (2) the evaluation of the effect of complexation on the dissolution and permeability of SUM-BCD and SUM-HPBCD complexes through an artificial membrane simulating gastrointestinal permeation as compared to the free form of SUM; (3) the pharmacological (*in vivo*) antiallodynic and antihyperalgesic activity of SUM-BCD and SUM-HPBCD complexes after their oral administration to mice as compared to both the free form of SUM and its commercially available ester form.

SUM-BCD and SUM-HPBCD inclusion complexes were obtained by co-grinding method. Their formation was a spontaneous, repeatable process. The presence of inclusion complexes was confirmed by using spectroscopic (Fourier transform infrared spectroscopy, FT-IR; solid state nuclear magnetic resonance spectroscopy with magic angle spinning condition, ^1^H and ^13^C MAS NMR) and thermal (differential scanning calorimetry, DSC) and methods.

**Figure 1:**
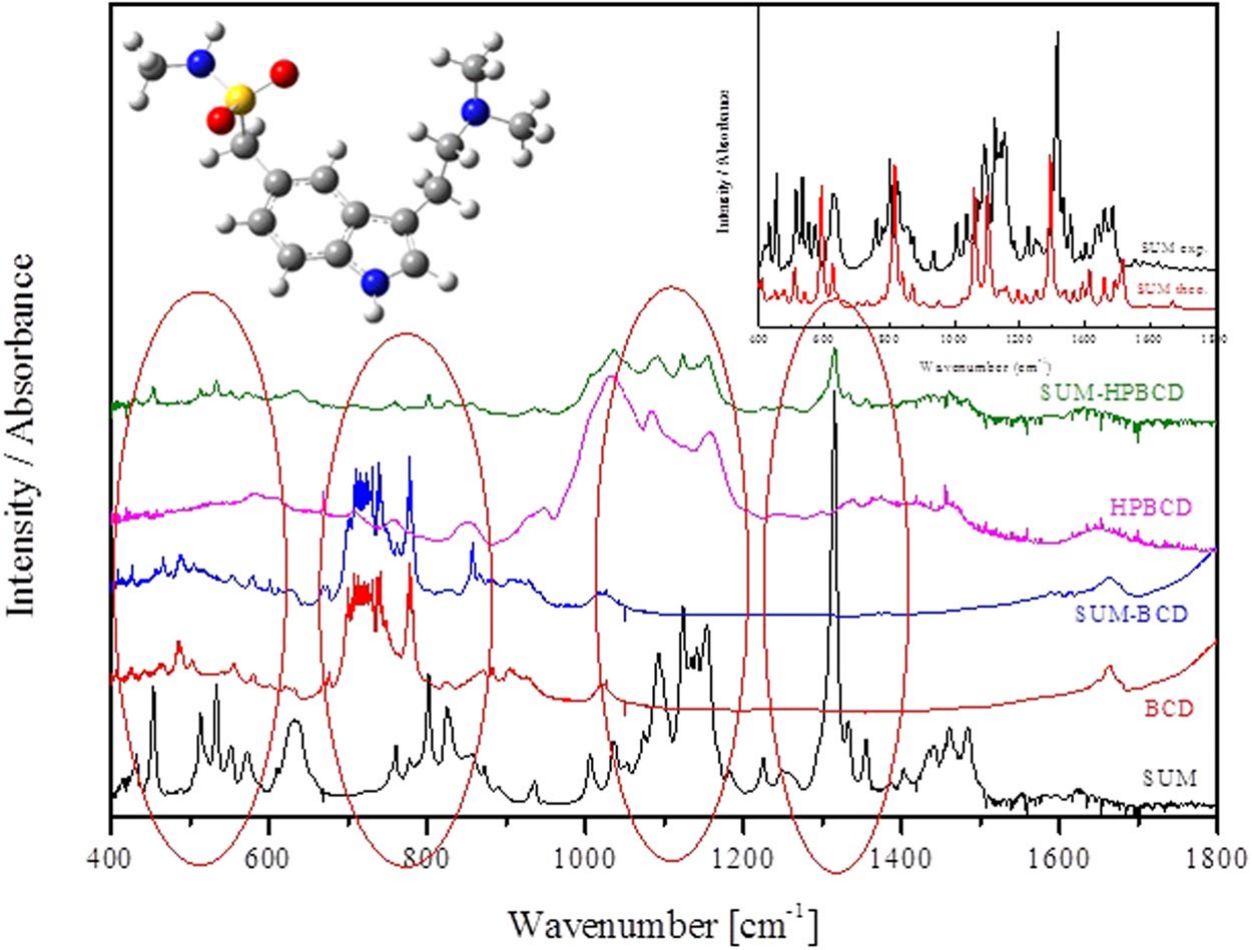
FT-IR absorption spectra for SUM, BCD, SUM-BCD, HPBCD and SUM-HPBCD inclusion complexes.

The types of bands, their location and intensity were determined for a SUM molecule in free form and compared with theoretical spectra based on quantum-chemical calculations performed with the use of the B3LYP functional and 6–31 G(d, p) as a basis set. A theoretical analysis, based on a simulation of molecular docking, suggested that, for the SUM-BCD and SUM-HPBCD complexes, inclusion occurred of an H-indol-5-yl structure and a dimethylaminoethyl group with a stronger retention of the phenyl ring in the HPBCD cavity (Fig. 1). The spectroscopic (FT-IR) analysis showed a marked decrease in the intensity of bands corresponding to an H-indol-5-yl domain or their disappearance in the following ranges corresponding to particular types of vibration and specific bonds: 600–850 cm^−1^, the bending vibrations of C-C-C; 640 cm^−1^, the wagging vibrations of C-H; 777 cm^−1^, the stretching vibrations of C-C and C-H; 830 cm^−1^, and the bending vibrations of C-C-H; 1400–1500 cm^−1^, the stretching vibrations of C-N; 1400–1500 cm^−1^, the deformation vibrations of C-C; 1392 cm^−1^, the bending vibrations of C-N-H; at 1457 cm^−1^, the skeleton vibrations of C-H; 1514 cm^−1^, the stretching vibrations of C-C and the wagging vibrations of C-H. The changes in band intensity in the range 1000–1300 cm^−1^ and the considerably reduced band intensity at 1460 cm^−1^ were attributed to the interaction of the dimethylaminoethyl group with the cyclodextrins. The most significant changes in band intensity were those corresponding to the wagging and twisting vibrations of the C-H bonds located at 1155 cm^−1^, 1183 cm^−1^ and 1333 cm^−1^. The band that differentiated the complexes of sumatriptan and the cyclodextrins relative to the dimethylaminoethyl group occurred at 1460 cm^−1^ and corresponded to the twisting vibrations of the C-H bond. In the SUM-HPBCD complexes, this band disappeared confirming the results of theoretical studies that suggested the stronger impact of the dimethylaminoethyl group toward the HPBCD cavity. All spectra are collected in Fig. 1. The changes described above were not observed for the physical mixture of SUM-BCD and SUM-HPBCD.

**Figure 2:**
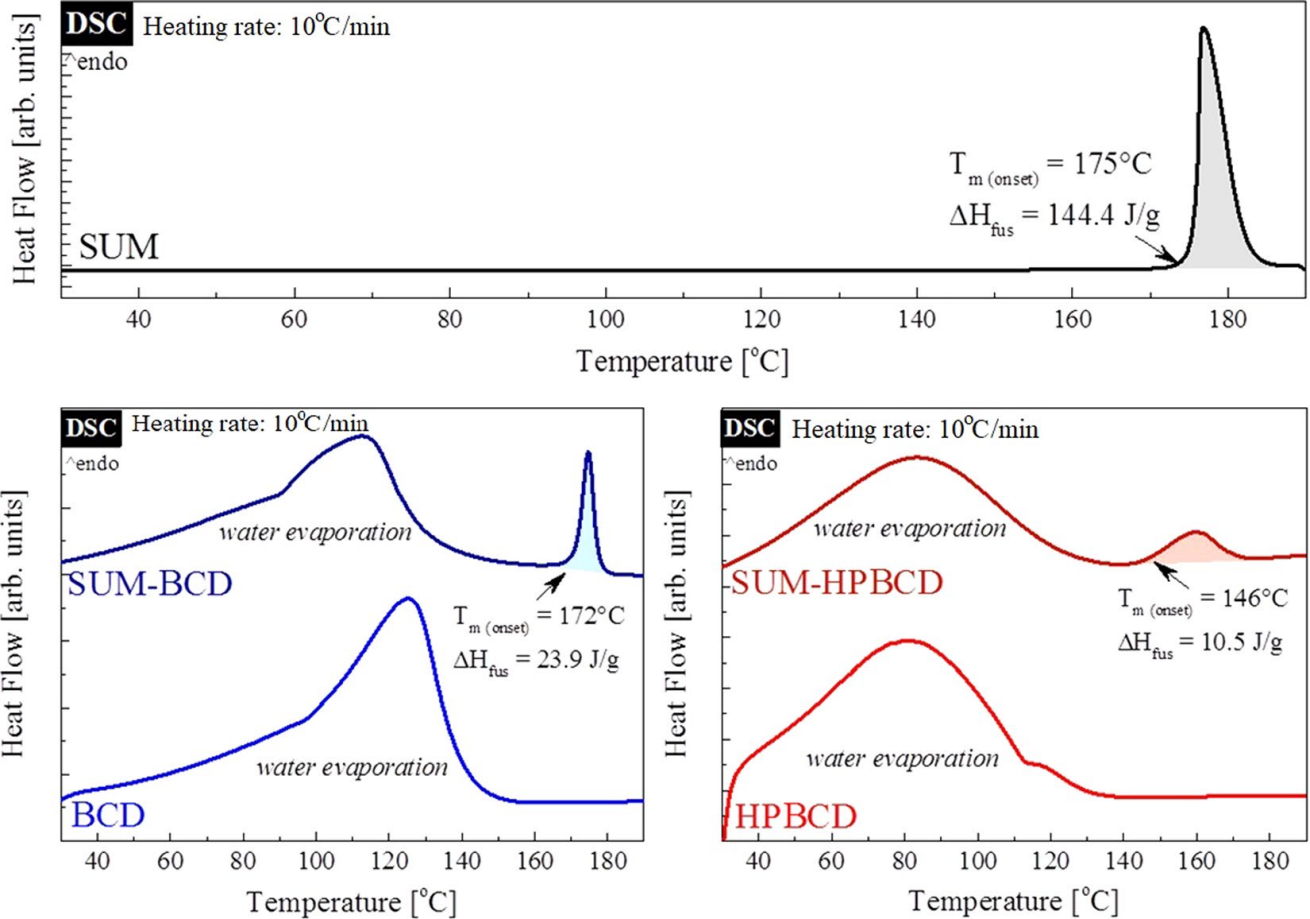
DSC thermograms of: neat SUM (upper panel); neat BCD (lower trace on the lower left panel); neat HPBCD (lower trace on the lower right panel); SUM-BCD (upper trace on the lower left panel); SUM-HPBCD (upper trace lower right panel).

Thermal properties of SUM, BCD, HPBCD, SUM-BCD and SUM-HPBCD were investigated by DSC. This technique enables the determination of presence or absence of the drug-melting endotherm, which might finally verify if the active pharmaceutical ingredient (API) was successfully complexed in CDs^24,25^. The DSC thermograms (obtained during heating–HR = 10 °C min^−1^) of SUM, BCD, HPBCD and binary systems of SUM-BCD and SUM-HPBCD are shown in Fig. 2. As can be seen, neat SUM exhibits a melting peak with an onset at 175 °C (ΔH_fus_ = 144.4 J g^−1^) which is typical for anhydrous crystalline drugs. The DSC traces of both BCD and HPBCD reveal a very broad endothermal peak located between 60 °C and 140 °C for BCD, and between 40 °C to 120 °C for HPBCD, related to the loss of water. In the case of the investigated formulations, i.e. SUM-BCD and SUM-HPBCD, two endothermic processes were registered during DSC measurements. The first, with the maximum located at 113 °C for SUM-BCD and at 83 °C for SUM-HPBCD, reflects water evaporation from the samples; and the second, smaller endothermic peak with an onset at 172 and 146 for SUM-BCD and SUM-HPBCD, respectively, corresponds to the melting point of SUM.

**Figure 3:**
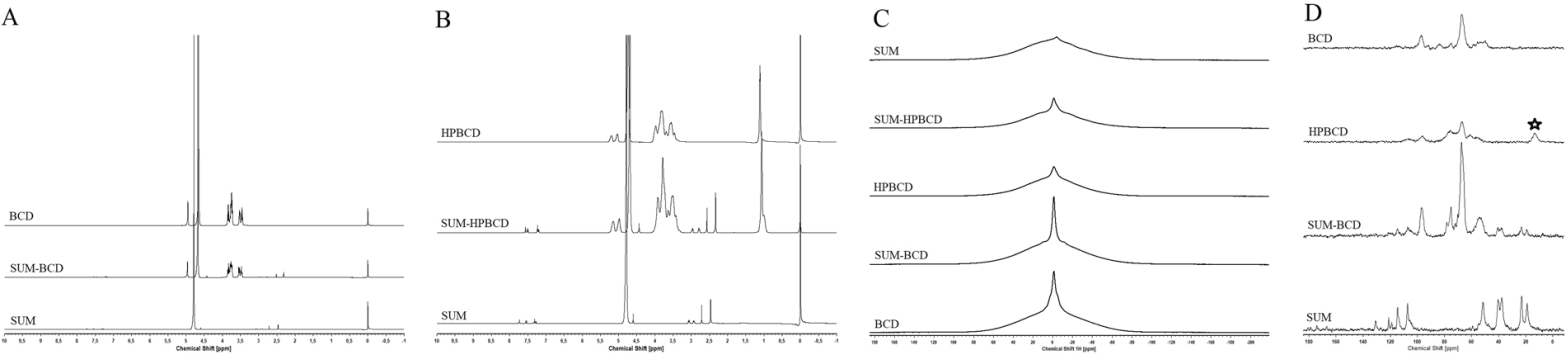
^1^H spectra in liquid (**A**,**B**), ^1^H spectra in solid (**C**) and ^13^C spectra in solid (**D**) of SUM, SUM-BCD inclusion complex, and SUM-HPBCD inclusion complex.

The solid state NMR with magic angle spinning (MAS) experiments were performed on two nuclei ^1^H and ^13^C. This extremely powerful method was widely used in studies of structure of complex macromolecular system in solid state including synthetic and natural polymers, surfactants as well as drug delivery systems^26^. The solid state NMR studies were recently used in the studies of some inclusion compounds based on cyclodextrin systems^27,28^. Therefore this method was selected as an additional tool to confirm the formation the sumatriptan/cyclodextrin inclusion compounds. The ^1^H and ^13^C NMR MAS spectra are presented in Fig. 3. In these comparison of spectra recorded for all studied complexes as well as reference substances are clearly visible characteristic changes in spectra of both complexes studied. These spectra are different from only superposition of signals from reference compound, which is a direct confirmation of formation of hydrogen bonds and formation of stable complexes. The most important changes are observed on the ^13^C spectrum in the ppm region from 50 to 60, where the broader band appeared. This band broadening ascribed to the formation of new rigid network as a result of interactions between cyclodextrin wall and included compound (SUM).

An HPLC-DAD method with the isocratic flow of the mobile phase was developed to determine the concentrations of sumatriptan during studies of dissolution profiles and permeability. The method was validated according to ICH guidelines^29^. Additionally, to confirm the absence of SUM degradation during the preparation of the inclusion complex, an HPLC-DAD method with a gradient mobile phase was applied, which permitted determination of all impurities^30^.

**Figure 4:**
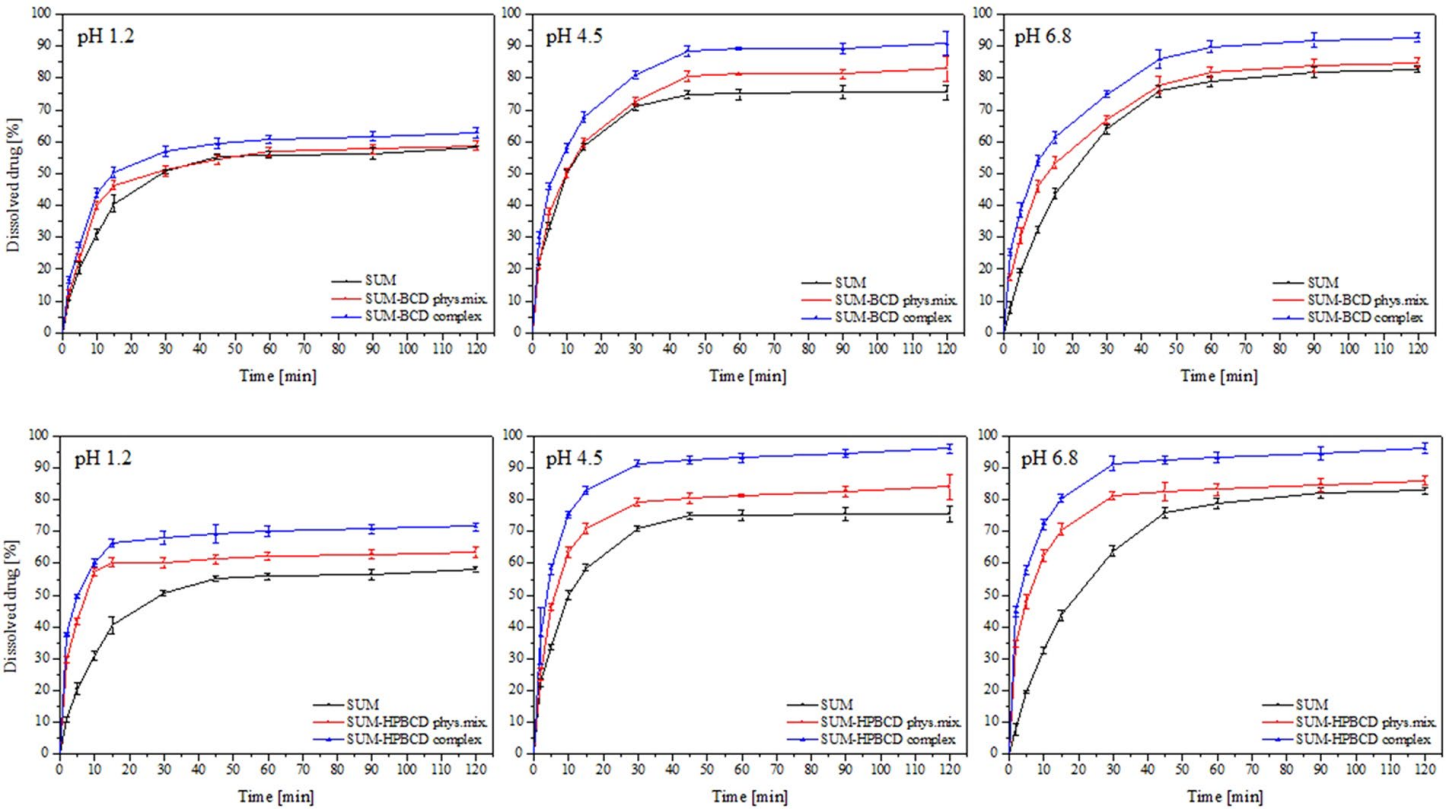
Dissolution profiles for SUM, SUM-BCD physical mixture, SUM-BCD inclusion complex, SUM-HPBCD physical mixture and SUM-HPBCD inclusion complex in artificial gastric juice at pH 1.2, in phosphate buffer at pH 4.5 and in phosphate buffer at pH 6.8.

The dissolution profiles of SUM and the SUM-BCD and SUM-HPBCD inclusion complexes were compared in simulated gastric fluids (pH 1.2) and phosphate buffer (pH 4.5 and 6.8) (Fig. 4). The similarity of the dissolution profiles of SUM, SUM-BCD and SUM-HPBCD was evaluated statistically on the basis of the Moore and Flanner model^31^. The similarity between the dissolution percentages of SUM in different forms was established based on parameters *f*_1_ and *f*_2_ and defined by the following equations:

$${f}_{1}=\frac{{\sum }_{j=1}^{n}|{R}_{j}-{T}_{j}|}{{\sum }_{j=1}^{n}{R}_{j}}\times 100$$

$${f}_{2}=50\times \,\mathrm{log}({(1+(\frac{1}{n})\sum _{j=1}^{n}{|{R}_{j}-{T}_{j}|}^{2})}^{-\frac{1}{2}}\times 100)$$

in which n is the number of withdrawal points, *R*_*j*_-the percentage of the dissolved reference compound at time point *t*, *T*_*j*_-the percentage of dissolved SUM-BCD or SUM-HPBCD at time point *t*.

According to this model and for all tested values of pH, the difference factor *f*_1_ exceeded 15 and the *f*_2_ factor was below 50, which reflected major differences between SUM, SUM-BCD and SUM-HPBCD complex dissolution profiles as well as the strong influence of cyclodextrins on the rate of SUM dissolution.

The second aspect of interest regarding the influence of cyclodextrins on SUM as a result of complexation comprised changes in the permeability of SUM through an artificial membrane in a model of the gastro-intestinal tract. This was assessed against a reference standard that was the permeability of SUM in free form. The apparent permeability coefficient (P_app_) was calculated from the following equation:

$${P}_{app}=\frac{-\,\mathrm{ln}(1-\frac{{C}_{A}}{{C}_{equilibrium}})}{S\times (\frac{1}{{V}_{D}}+\frac{1}{{V}_{A}})\times t}$$

where *V*_D_–donor volume, *V*_A_–acceptor volume, *C*_equilibrium_–equilibrium concentration ${C}_{equilibrium}=\frac{{C}_{D}\times {V}_{D}+{C}_{A}\times {V}_{A}}{{V}_{D}+{V}_{A}}$, *C*_D_–donor concentration, *C*_A_–acceptor concentration, *S*–membrane area, *t*–incubation time (in seconds).

**Figure 5:**
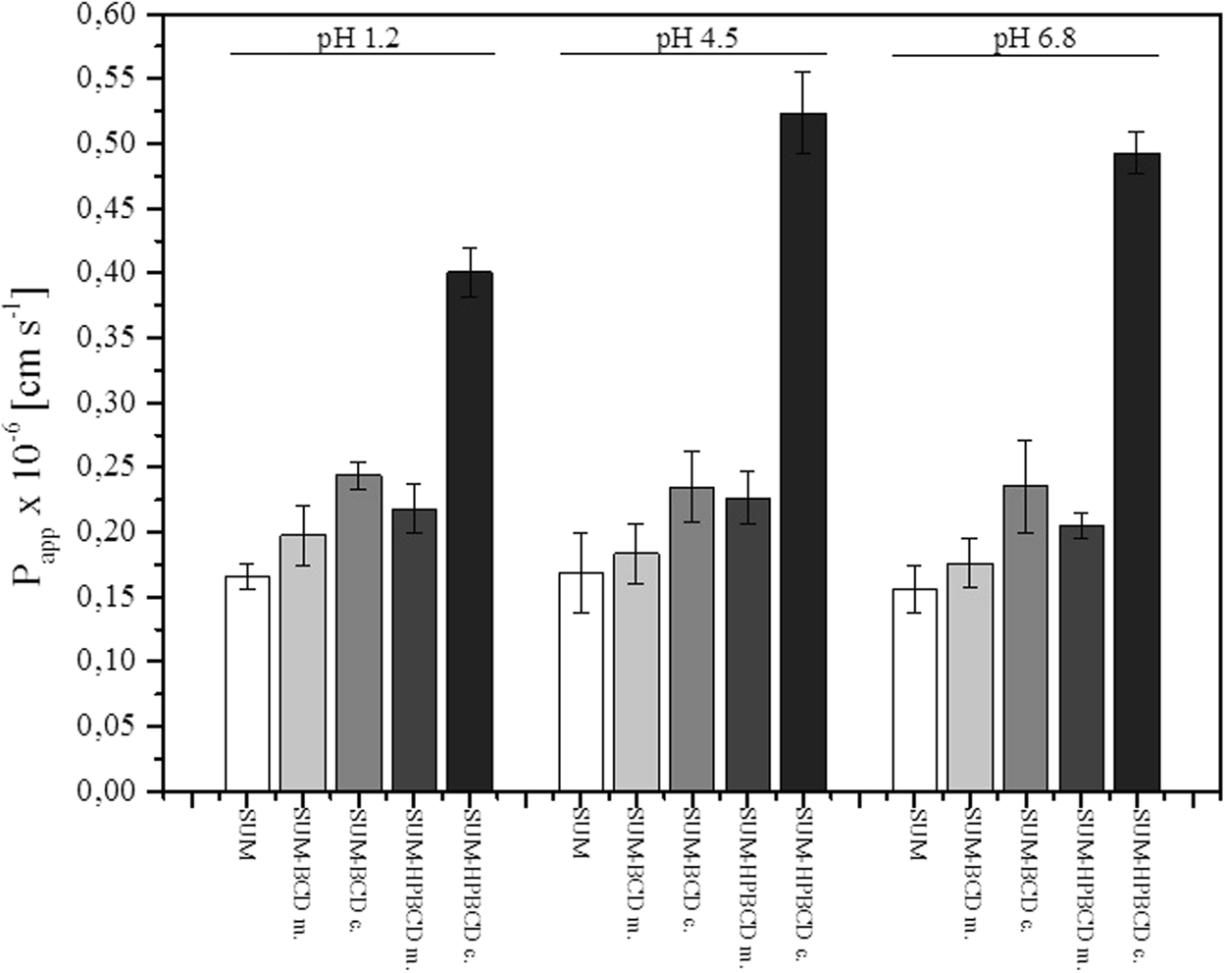
Apparent permeability coefficient for SUM, SUM-BCD physical mixture (abbr. “m.”), SUM-BCD complex (abbr. “c.”), SUM-HPBCD physical mixture and SUM-HPBCD complex in artificial gastric juice at pH 1.2, in phosphate buffer at pH 4.5 and in phosphate buffer at pH 6.8.

The permeability of SUM in complexed forms (SUM-BCD and SUM-HPBCD) and in free form was studied for the gastrointestinal tract using a PAMPA system at three values of pH: 1.2, 4.5 and 6.8 (Fig. 5). Apparent permeability coefficients were found for all systems under study. To verify that P_app_ determined for permeability was statistically different, an ANOVA test was used. Compounds with P_app_ < 1 × 10^−6^ cm s^−1^ are classified as low-permeable and those with P_app_ > 1 × 10^−6^ cm s^−1^ as high-permeable compounds. All systems showed low values of apparent permeability coefficients under all study conditions. Following the formation of complexes with cyclodextrins, a statistically significant improvement was found in the permeability of SUM through the membrane structures of the gastrointestinal tract model (PAMPA). Increased permeability was observed in all acceptor systems. A twofold rise in permeability occurred during the inclusion of SUM into HPBCD ((0.16 ± 0.01) × 10^– 6^ cm s^−1^ and (0.40 ± 0.02) × 10^−6^ cm s^−1^ at pH 1.2, (0.17 ± 0.03) × 10^–6^ cm s^−1^ and (0.52 ± 0.03) × 10^−6^ cm s^−1^ at pH 4.5, (0.16 ± 0.02) × 10^–6^ cm s^−1^ and (0.49 ± 0.02) × 10^−6^ cm s^−1^ at pH 6.8 for SUM and SUM-HPBCD complexes, respectively).

As a result of a considerable increase in the solubility and permeability of SUM after its incorporation into the cyclodextrins systems, a decision was taken to continue the study in an *in-vivo* model, where an additional reference (alongside SUM in free form) was a study of the analgesic effect of sumatriptan succinate available in commercial preparations.

**Figure 6:**
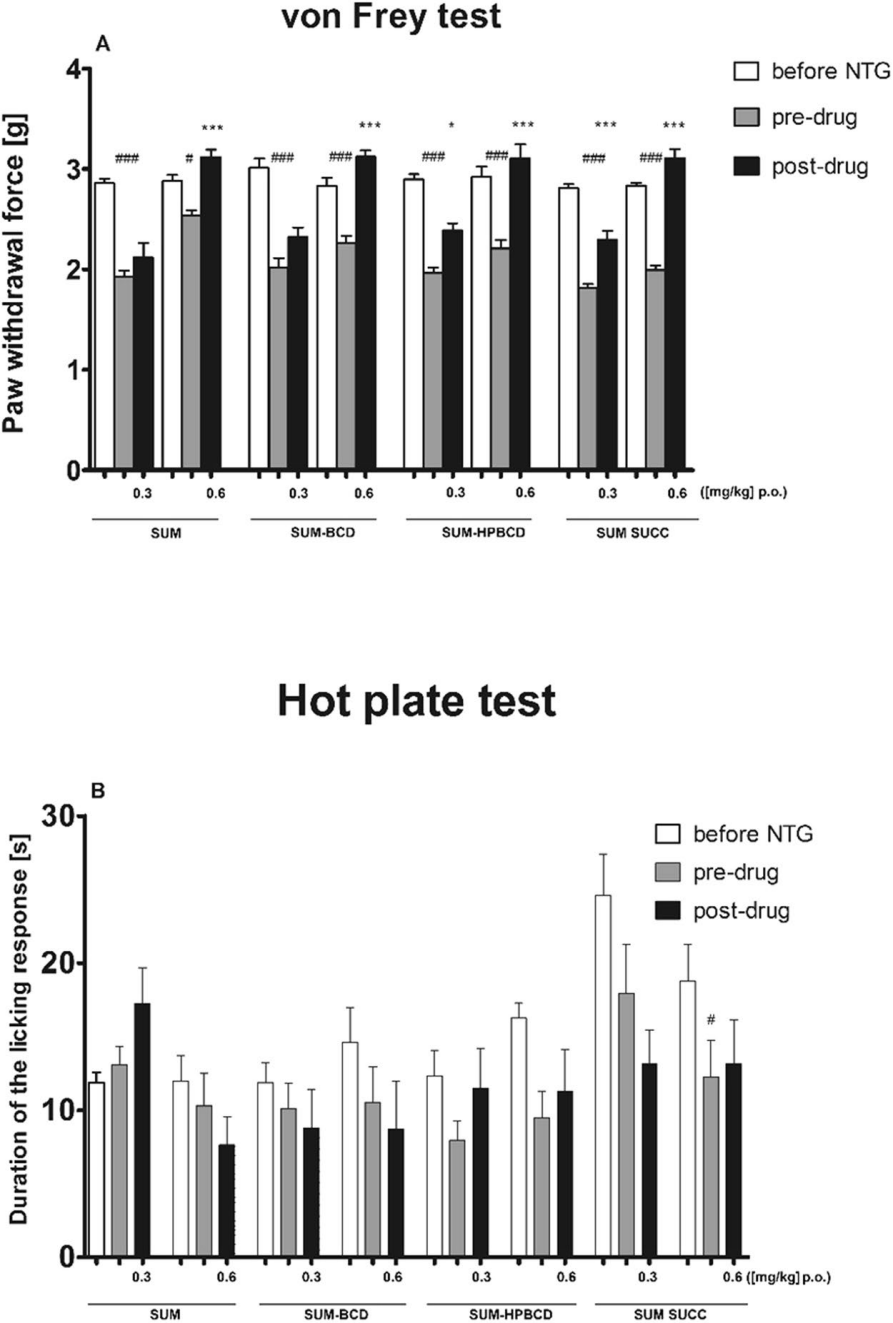
Influence of SUM forms on tactile allodynia (**A**) and thermal hyperalgesia (**B**) in a mouse model of migraine pain induced by nitroglycerin (NTG) administration. Results are shown as mean paw withdrawal forces (±SEM) measured in the von Frey test (**A**), or latency time to pain reaction (±SEM) measured in the hot plate test (**B**) for n = 8–10. Statistical analysis: analysis of variance (ANOVA), followed by Tukey’s *post hoc* test. Significance *vs*. baseline (i.e. before nitroglycerin administration) paw withdrawal force, or latency time to pain reaction: ^#^p < 0.05, ^###^p < 0.001. Significance *vs*. pre-drug (i.e. before SUM form administration) measurement: *p < 0.05, ***p < 0.001.

During *in vivo* studies in the von Frey test, an overall effect of treatment was observed for sumatriptan (F[5, 54] = 35.60, p < 0.001). The dose 0.6 mg kg^−1^ attenuated tactile allodynia significantly (p < 0.001), but a lower dose was not active (Fig. 6A). An overall effect of treatment was also observed for sumatriptan-BCD (F[5, 51] = 30.53, p < 0.0001). *Post hoc* analysis revealed that the antiallodynic effect was significant (p < 0.001) only for the dose of 0.6 mg kg^−1^ (Fig. 6A). SUM-HPBCD and sumatriptan succinate significantly affected tactile allodynia in this pain model (F[5, 48] = 23.04, p < 0.001; F[5, 54] = 79.57, p < 0.001, respectively). Statistically significant antiallodynic properties were demonstrated for both doses of these two sumatriptan forms (Fig. 6A).

In the hot plate test, an overall effect of treatment was observed for sumatriptan (F[5, 51] = 3.082, p < 0.05). However, *post hoc* analysis did not show significant differences between measured pre-drug and post-drug latencies (Fig. 6B). For SUM-BCD and SUM-HPBCD, no overall effect of treatment was observed in the hot plate test (F[5, 54] = 0.88, p > 0.05 for SUM-BCD and F[5, 45] = 1.900, p > 0.05 for SUM-HPBCD, Fig. 5B). In this assay, an overall effect of treatment was observed for sumatriptan succinate (F[5, 48] = 3.00, p < 0.05), but no significant differences between pre-drug and post-drug latencies were observed for either dose of this drug form (Fig. 6B).

**Figure 7:**
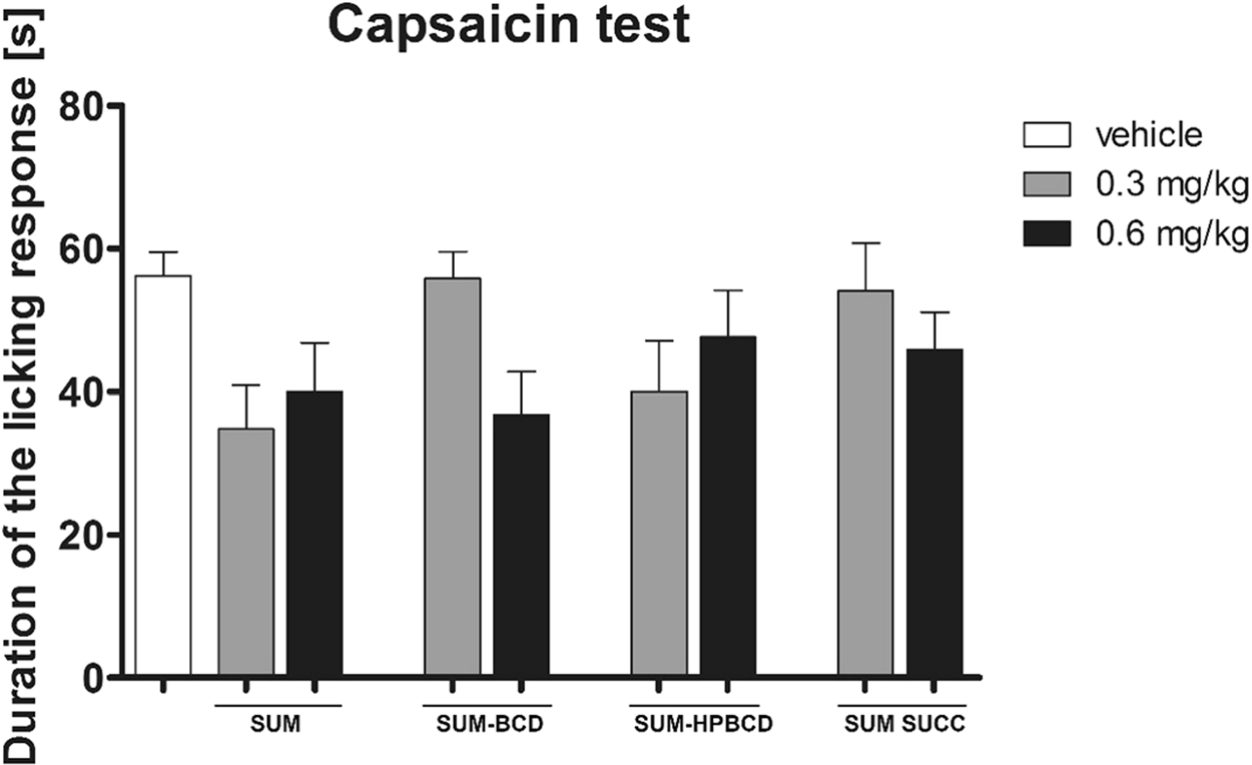
Antinociceptive activity of SUM forms in the capsaicin-induced neurogenic pain model in mice. Results are shown as duration of the licking/biting response (±SEM) for n = 6–9. Statistical analysis: one-way analysis of variance, followed by Dunnett’s *post hoc* comparison. Results were not significant (p > 0.05).

In the neurogenic pain model induced by capsaicin administration, an overall effect of treatment was observed (F[8, 53] = 2.152, p < 0.05), but the *post hoc* analysis of results obtained for drug-treated groups compared to vehicle-treated mice showed no significant differences between groups (Fig. 7).

## Discussion

The inclusion complexes SUM-BCD and SUM-HPBCD (1:1) were obtained by co-grinding method. The inclusion was a spontaneous process requiring no use of organic solvents, which eliminated the need to dispose of or control their volatile residues. Equally importantly, such a process is easily scalable. A similar phenomenon of spontaneous and permanent inclusion has been observed for other active pharmaceutical substances^32,33^. Spectroscopic (FT-IR, NMR) and thermal (DSC) methods were used to confirm the identity of the complexes. The use of the FT-IR method based on a theoretical approach to the determination of theoretical spectra and the application of docking to propose the domains involved in the interaction with the cyclodextrins made it possible to accurately obtain the SUM structures directly involved in interactions with the inside of a cyclodextrin cavity. Hydrophobic domains such as the H-indol-5-yl structure and the dimethylaminoethyl group were indicated as the incorporated ones. During interactions with HPBCD, the dimethylaminoethyl group was more involved in interaction with the cyclodextrin cavity.

Thermal analysis of the investigated systems reveals that both SUM-BCD and SUM-HPBCD formulations possess some free SUM (not trapped inside CD). This is evidenced by the presence of the small peak with an onset at 172 °C and 146 °C for SUM-BCD and SUM-HPBCD, respectively. These endothermal peaks originate from the melting of free SUM existing in the examined binary compositions. From the comparison of the fusion enthalpies of the neat API and the mixtures, it was possible to estimate the degree of CD inclusion. Knowing (1) the molecular weight of all components as well as (2) the weight percentage of SUM in the formulations, one might calculate the enthalpy of fusion of the ideal systems in which there is no inclusion (29.8 J g^−1^ and 25.2 J g^−1^ for SUM-BCD and SUM-HPCD, respectively). Comparing these–calculated–values to the experimental data, it was possible to estimate that, in the SUM-BCD formulation, 19.8% of SUM is trapped inside BCD, while in the SUM-HPBCD formulation CD inclusion by SUM is equal to 58.3%.

As presented in Fig. 3 the characteristic changes in NMR spectra, especially in ^13^C NMR, the formation of inclusion compound has been confirmed. The broadened band at 60 ppm exhibits the formation of hydrogen bond to the cyclodextrin OH groups and hindered or even blocked the rotation of CH_2_ group located between sulfodixide and H-indol-5-yl structure fragments of SUM.

According to the dissolution profiles, the inclusion complexes SUM-BCD and SUM–HPBCD demonstrated superior release characteristics in simulated intestinal fluids for all pH values (1.2, 4.5 and 6.8). In the case of SUM-BCD and SUM-HPBCD, the slightest improvement in solubility was registered at pH 1.2. Under such conditions, SUM was also characterized by the lowest solubility. The considerably better solubility of both cyclodextrin complexes was observed and resulted in the different shapes of their dissolution profiles. The increased solubility of sumatriptan could be explained by the inclusion into the cyclodextrins of its two hydrophobic domains: the H-indol-5-yl structure and the dimethylaminoethyl group. The presence of a greater number of hydrophilic groups on the surface of HPBCD was responsible for the superior solubilization of sumatriptan in the case of its inclusion into the complex. This process was intensified by the stronger retention of the dimethylaminoethyl group inside the HPBCD complex. These results correspond to those published in reports on the use of cyclodextrins as release modifiers for certain molecules, which have confirmed the significant potential of cyclodextrins as excipients in this respect^34,35^. For example, the solubility of naringenin has been found to increase twofold after its inclusion into a cyclodextrin^36^. In the case of capsaicin, the rise in solubility following inclusion into HPBCD is markedly greater compared to its interactions with BCD^35^.

The permeability experiments also showed significant differences in the apparent permeability coefficient for complexed SUM in relation to its free form. An approximately 25% increase was observed for the SUM-BCD complexes and an over twofold rise within the entire pH range studied for SUM-HPBCD. As demonstrated by Warner, SUM is absorbed throughout the gastrointestinal tract and its absorption is similar after oral and jejunal administration, and less after cecal administration^37^. The increased permeability of sumatriptan is most certainly a consequence of its greater solubility due to inclusion into cyclodextrins. According to Loftsson, the improved dissolution of drugs in aqueous gastrointestinal fluids causes diffusion toward the mucosal surface and better immediate absorption^38^. Enhanced bioavailability resulting from the formation of inclusion complexes has been reported for a number of drugs whose poor solubility reduced their absorption^39^. Limited functionality of cyclodextrins in relation to compounds requiring transport systems for absorption through biological membranes that rely on phenomena other than passive diffusion as well as the lower bioavailability of drugs in which inclusion comprises their hydrophobic domains^40^. There have been many reports indicating that the inclusion of a drug into a cyclodextrin enhances its bioavailability^41^. For instance, acyclovir in a complex with hydroxypropyl-β-cyclodextrin has shown promising results regarding oral therapy for the treatment of herpes viruses^42^. With respect to the present work, the increased solubility of sumatriptan achieved by inclusion complexation with cyclodextrins ensured the drug’s better dissolution in all regions of the gastrointestinal tract, resulting in greater permeability and, probably, bioavailability. Previous studies have demonstrated that sumatriptan is absorbed poorly but rapidly, which explains the enhancement of permeability for all pH values studied as a result of an increase in the concentration of dissolving SUM over a short period of time in the case of cyclodextrin systems^43^. The bioavailability of sumatriptan upon oral administration is also limited by the effect of first-pass metabolism and amounts to approx. 14% (Scott 1994). Efforts to eliminate the aforesaid effect have included the development of intranasal and buccal forms of sumatriptan succinate^44,45^. Shivanand demonstrated the possibility of increasing the bioavailability of sumatriptan succinate following buccal administration^45^. The literature also refers to enhancement of the bioavailability of some active substances due to the part played by cyclodextrin complexes in the saturation of enzymes involved in the first-pass metabolism. Substances in cyclodextrin complexes have been found to dissolve more rapidly. Therefore, their absorption can lead to enzyme saturation during their first pass through the liver, resulting in a higher fraction of unmetabolized drugs entering the systemic circulation compared to the situation with substances in free form with a lower dissolution rate. The results obtained within the scope of the present study, indicating an improvement in the solubility of sumatriptan and an increase in its permeability through artificial membranes simulating the gastrointestinal tract across the whole pH range, formed the basis for further experiments on an animal model.

In the *in vivo* part of the present study, we compared anti-migraine pain properties of two novel forms of SUM complexed with cyclodextrins (SUM-BCD and SUM-HPBCD) with classic sumatriptan in a base form and sumatriptan succinate. To assess the antiallodynic and antihyperalgesic properties of these orally administered forms of sumatriptan, we used a mouse model of migraine pain induced by nitroglycerin. Nitroglycerin is a donor of nitric oxide which is a molecule implicated in migraine pathogenesis and migraine-associated hypersensitivity^46^. In laboratory animals, intraperitoneally administered nitroglycerin may trigger migraine-like pain, so it could induce key behavioral responses (tactile allodynia and thermal hyperalgesia) that resemble migraine symptoms in humans. The dose of nitroglycerin was chosen based on previous reports that demonstrated a rapid development of allodynia without any undesirable effect on systemic blood pressure^34^.

During the headache phase in migraine, mechanical allodynia is the most prominent symptom and stimuli that are normally innocuous are detected as painful. Migraineurs experience increased sensitivity to non-noxious thermal and mechanical stimulation and cutaneous allodynia in these patients is a clinical manifestation of central nervous system sensitization^47^. Migraine-related skin hypersensitivity occurs both in trigeminal and at extracephalic sites^19^. Therefore, in our *in vivo* research we focused on the effect of sumatriptan forms on tactile allodynia and we used the von Frey test as a well-established assay to measure antiallodynic properties of the drugs tested. In line with previous studies, the injection of nitroglycerin induced mechanical allodynia which was sensitive to acute migraine therapy using all forms of sumatriptan; however, it was only for sumatriptan-HPBCD and sumatriptan succinate that the dose of 0.3 mg kg^−1^ was effective.

Our present research that utilized nitroglycerin failed to establish any effect of sumatriptan forms on heat hyperalgesia. There might be several reasons to explain the inability of nitroglycerin to induce thermal hypersensitivity. Firstly, in contrast to other authors who tested sumatriptan on C57BL/6J mice, we used a distinct mouse strain^48^. Secondly, the hot plate test is an assay for the detection of pain of central origin and it assesses nocifensive responses at the supraspinal level, so it might not be able to reveal peripheral pain responses^49^. Thermal hyperalgesia in migraine models might be related to peripheral inflammation and sumatriptan was able to attenuate thermal hypersensitivity induced by intraplantar injection of carrageenan but it had no effect on thermal hyperalgesia caused by sciatic nerve injury^50^. This differential effect might be explained in terms of the distinct responses of sensory neurons in inflammatory and neuropathic pain conditions, which leads to distinct mechanisms that underlie the development of thermal hyperalgesia in these two pain states.

To further investigate the anti-migraine properties of sumatriptan forms, we implemented the capsaicin test. The role of TRPV1 and capsaicin-sensitive fibers in migraine is still controversial, but it has been demonstrated that capsaicin is a potent stimulant of the trigeminovascular system and is responsible for the release of CGRP, a key mediator in migraine pain^51^. The release of CGRP from capsaicin-sensitive trigeminal sensory nerves during migraine attacks results in cranial vasodilatation and central nociception; hence, trigeminal inhibition may prevent this vasodilatation and attenuate migraine headache^52^. The anti-migraine properties of triptans may be due to vasoconstriction of the carotid arterial bed via 5-HT_1B_ receptors and the inhibition of CGRP release from trigeminal nerves via 5-HT_1B/D_ receptors, in our study sumatriptan forms were inactive in the capsaicin test. This lack of efficacy of sumatriptan in this test is, at least to some degree, in line with the results obtained by other authors who have shown that intravenous sumatriptan is not effective in the inhibition of capsaicin-induced responses related to cranial vasodilatation and central nociception^53^.

## Experimental

### Materials for *in vitro* studies

Sumatriptan and sumatriptan succinate (purity > 98%) were supplied by Pharmachem International Co. (China). β-Cyclodextrin (purity > 98%) and 2-hydroxylpropylo-β-cyclodextrin were obtained from Sigma-Aldrich (Poland). Acetonitrile of UHPLC grade was supplied by Merck KGaA (Germany). Formic acid (100%), hydrochloric acid and potassium dihydrogen phosphate were obtained from Avantor Performance Materials (Poland). High-quality pure water was prepared using a Millipore Exil SA. 67120 purification system.

### Preparation of the cyclodextrin inclusion complex

The solid inclusion complex of sumatriptan (SUM) with β-cyclodextrin (BCD) and 2-hydroxylpropylo-β-cyclodextrin (HPBCD) was obtained by the co-grinding method. Co-ground SUM-BCD and SUM-HPBCD were prepared by ball-milling the SUM and BCD or HPBCD in the same molar ratio 1:1 in a ball mill (Retsch MM400) at a frequency of 30 Hz for 60 minutes. The cyclodextrin complexes were kept in an atmosphere of controlled humidity at 45% RH.

### Characterization of the SUM-BCD and SUM-HPBCD inclusion complexes

During identification studies of the SUM-BCD and SUM-HPBCD complexes, spectroscopic (FT-IR, NMR) and thermal (DSC) techniques were used. An HPLC-DAD method was applied for the measurement of SUM concentration changes during studies of dissolution and permeability through biological membranes.

#### Spectroscopic analysis

SUM and SUM-BCD and SUM-HPBCD inclusion complexes were obtained separately with IR grade potassium bromide at the ratio of 1:100, and IR pellets were prepared by applying 8 metric tons of pressure in a hydraulic press. The vibrational infrared spectra were measured between 500 and 1800 cm^−1^ with an FT-IR Bruker Equinox 55 spectrometer equipped with a Bruker Hyperion 1000 microscope. In order to analyze positions and intensity of bands in experimental spectra of SUM, quantum-chemical calculations were performed based on B3LYP functional and 6–31 G(d, p) as a basis set. All the calculations were made using the Gaussian 09 package and the GaussView application^54^. While for prediction of the interactions of SUM and CD, theoretical research was conducted which presented the most provisional places of SUM inclusion into the cyclodextrin cavity.

#### Theoretical analysis

Theoretical studies were conducted with the application of docking and molecular modeling approaches. The SUM as well as macromolecule geometries were optimized using Density Functional Theory (DFT) with a hybrid functional B3LYP (Becke, 3-parameter, Lee-Yang-Parr) and 6–31 G(d, p) basis set. AutoDock Vina was used to find optimal binding modes of SUM–CD inclusion complexes. The geometries of most energetically favored binding modes were fine-tuned by a search for the lowest energy in respect to ligand rotation inside the CD cavity. Parameterized Method 6 was used to optimize geometry in systems with ligands rotated in a range from 0–180°. The binding energy of the most energetically favored binding mode at PM6 level of theory was calculated with the DFT B3LYP 6–31 G(d, p) method with Basis Set Superposition Error correction.

#### Thermal analysis

Thermal properties of neat SUM, BCD, HPBCD as well as inclusion complexes SUM-BCD and SUM-HPBCD were examined using a Mettler-Toledo DSC 1 STARe System. The measuring device was calibrated for temperature and enthalpy using zinc and indium standards. The instrument was equipped with an HSS8 ceramic sensor with 120 thermocouples and a liquid nitrogen cooling station. Samples of about 5 mg were measured in an ehaviour crucible (40 μL). All measurements were performed in a range from 30 °C to 190 °C with a heating rate equal to 10 °C min^−1^.

#### NMR analysis

The NMR studies were performed by the use of NMR Agilent 400 MHz spectrometer and Wide Bore Triple Resonance T3 MAS XY{1H-19F} probe head. ^13^C experiment was performed using the standard one pulse sequence with the decoupler mode with TPPM (two pulses phase modulation) schema. For each sample, loaded in ZrO_2_ 4-mm spinner, the NMR spectrum collected was a result of NS = 1024 accumulations, collected every 60 seconds with the spinning rate 5 kHz. ^1^H solid-state NMR spectra were recorded in the same conditions with single pulse sequence of NS = 16 and repetition time 60 s.

### Studies of the physicochemical properties of the SUM-BCD and SUM-HPBCD inclusion complexes

The changes in SUM concentrations during dissolution and permeability through artificial biological membranes of the gastro-intestinal tract were determined using the HPLC-DAD method. The HPLC-DAD method with an isocratic mobile phase was developed and validated. The determination of SUM was possible using an LC system (Dionex Thermoline Fisher Scientific, Germany) equipped with a high pressure pump (UltiMate 3000), an autosampler (UltiMate 3000) and a DAD detector (UltiMate 3000) with Chromeleon software version 7.0 from Dionex Thermoline Fisher Scientific (US). Separations were performed on a Kinetex C-18 (100 mm × 2.10 mm, 2.6 µm) column. The detection of SUM was performed using a diode array detector at a wavelength maxima (𝜆_max_) of 282 nm. The mobile phase consisted of a mixture of 0.1% formic acid and acetonitrile (80:20 V/V) with a mobile phase flow rate of 0.5 mL min^−1^. The column was set at 30 °C. For determination of the impurities of SUM which can be formed during the formation of its cyclodextrins complexes, HPLC-DAD was applied with a gradient mobile phase.

### Dissolution studies of SUM and SUM-BCD and SUM-HPBCD inclusion complexes

Dissolution studies of SUM from SUM-BCD and SUM-HPBCD inclusion complexes were performed using a standard paddle Agilent 708-DS Dissolution Apparatus with a 500-mL dissolution medium at 37 ± 0.5 °C and 50 rpm for 120 min. Samples were weighted into gelatin capsules and then were placed in a spring to prevent flotation of the capsule on the surface of the liquid. As dissolution media, artificial gastric juice at pH 1.2 and phosphate buffer at pH 4.5 and 6.8 were used. At appropriate time intervals, dissolution samples (5.0 mL) were collected with the replacement of equal volumes of temperature-equilibrated media. Samples were then filtered through a 0.45 μm membrane filter.

#### Permeability studies of SUM and SUM-BCD and SUM-HPBCD inclusion complexes

Differences in the gastrointestinal permeability of SUM, SUM-BCD and SUM-HPBCD physical mixtures, and SUM-BCD and SUM-HPBCD complexes were investigated by using a PAMPA method (parallel artificial membrane permeability assay). The system consisted of a 96-well microfilter plate and a 96-well filter plate and was divided into two chambers: a donor at the bottom and an acceptor at the top, separated by a 120-μm-thick microfilter disk coated with a 20% (w/v) dodecane solution of a lecithin mixture (Pion, Inc.). The donor solution was adjusted to pH 1.2, 4.5 and 6.8. The SUM, SUM-BCD and SUM-HPBCD were dissolved in the donor solution. The plates were put together and incubated at 37 °C for 2 h in a humidity-saturated atmosphere. The concentrations of SUM, SUM-BCD and SUM-HPBCD in the donor and acceptor compartments were determined using the HPLC-DAD method.

### *In vivo* studies

#### Chemicals used for *in vivo* assays

For *in vivo* tests, the compounds were prepared in 1% Tween 80 solution (Polskie Odczynniki Chemiczne, Poland) and they were administered by the oral route. To perform quantitative comparison of the antiallodynic, antihyperalgesic and antinociceptive activities of drugs to be tested, we selected two doses for each form of sumatriptan. These doses corresponded to those found in the available literature about sumatriptan (0.3 and 0.6 mg kg^−1^, oral route). Control mice received 1% Tween 80. Nitroglycerin (Perlinganit, 1 mg 1 mL^−1^, UCB Pharma GmbH, Germany) was administered by the intraperitoneal route 45 min before ehavioural tests. Capsaicin was provided by Sigma Aldrich (Poland). For the *in vivo* experiments, this capsaicin was dissolved in ethanol (100%) at 5% of the final desired volume and then 0.9% saline was added. This mixture was vortexed for 10 min^55^.

#### Animals and housing conditions

In *in vivo* tests, adult male Albino Swiss (CD-1) mice weighing between 18–22 g were used. The animals were purchased from the Animal Breeding Farm of the Jagiellonian University in Kraków. The animals were housed in groups of 10 mice per cage at a room temperature of 22 ± 2 °C, under a light/dark (12:12) cycle. The animals had free access to food and tap water before experiments. Both the ambient temperature of the experimental room and humidity (50 ± 10%) were kept constant throughout all the tests. For ehavioural experiments, the animals were selected randomly. Each experimental group consisted of 6–10 animals/dose. The experiments were performed between 9:00 AM and 2:00 PM. Immediately after the *in vivo* assays, the animals were euthanized by cervical dislocation. All procedures were approved by the Local Ethics Committee of the Jagiellonian University in Kraków (7.12.2017) and the treatment of animals was in full accordance with ethical standards laid down in respective Polish and EU regulations (Directive No. 86/609/EEC).

#### Nitroglycerin-induced migraine-like pain model

Induction phase: In order to develop migraine-like pain in mice, the animals were injected intraperitoneally with a single dose of nitroglycerin (10 mg kg^−1^)^56^. To assess the influence of the test drugs on tactile allodynia and heat hyperalgesia, the pain sensitivity threshold of experimental animals was assessed using the von Frey test and the hot plate test, respectively. Measurements were collected before nitroglycerin administration (referred to as “before nitroglycerin”), 45 min after its injection (baseline, pre-drug measurement) and then 45 min after sumatriptan administration (post-drug measurement).

Influence on tactile allodynia (von Frey test): Mechanical hypersensitivity (tactile allodynia) in mice was assessed using an electronic von Frey unit (Panlab, Spain) supplied with a single flexible filament applying increasing force (from 0 to 10 g) against the plantar surface of the hind paw of a mouse. The nocifensive paw withdrawal response automatically turned off the stimulus and the mechanical pressure that evoked the response was recorded. On the day of the experiment, the mice were placed in individual test compartments with a wire mesh bottom and were allowed to habituate for 30 min. After the habituation period, each mouse was tested 3 times alternately in each hind paw, allowing at least 30 s between each measurement. Then, the mice were pretreated with nitroglycerin and 45 min later the animals were tested again for their pain sensitivity threshold (pre-drug measurement)^57^. This procedure was also repeated 45 min after sumatriptan administration (post-drug measurement).

#### Influence on thermal hyperalgesia (hot plate test)

Immediately after the von Frey test, the antihyperalgesic properties of sumatriptan forms were assessed in the hot plate test according to a slightly modified method as previously described^58^. The hot plate apparatus (Hot/cold plate, Bioseb, France) is supplied with a temperature-controller that maintains surface temperature to a set point (here 55–56 °C). In this assay, the animals were first tested to obtain baseline latencies to pain reaction (licking hind paws or jumping) before nitroglycerin injection (referred to as latencies “before nitroglycerin”). Then, nitroglycerin was injected and latencies to pain reactions were measured again (referred to as “pre-drug latencies”). Subsequently, test drugs were administered and post-drug latencies were measured again. In this assay, a cut-off time of 60 s was established to avoid potential paw tissue damage and animals not responding within 60 s were removed from the apparatus and assigned a score of 60 s.

#### Capsaicin-induced neurogenic pain model

Capsaicin tests were performed 60 min after the administration of test compounds or vehicle. After the adaptation period (15 min), 1.6 μg of capsaicin dissolved in 20 μL of a mixture containing 0.9% saline and ethanol (5% of the final volume) was injected intraplantarly in the ventral surface of the right hind paw of each mouse. The animals were observed individually for 5 min following capsaicin injection. In all experimental groups, the duration of the nocifensive behavior, i.e. the amount of time spent on licking, biting or lifting the injected paw, was recorded with a chronometer and this was compared between drug-treated and vehicle-treated groups^52^.

#### Data analysis

Data analysis of the results obtained in behavioral tests was provided by GraphPad Prism Software (v.5, CA, USA). The results were statistically evaluated using analysis of variance (ANOVA), followed by Tukey’s or Dunnett’s *post hoc* comparisons. P < 0.05 was considered significant.

## Conclusion

The inclusion of sumatriptan into cyclodextrin complexes increased its solubility and, consequently, improved its permeability through the biological membranes of a gastrointestinal tract model across an entire pH range of 1.2–6.8. Changes in the physicochemical parameters of sumatriptan were reflected in its enhanced pharmacological *in vivo* activity in terms of the assessment of migraine pain and attendant symptoms. The antiallodynic action of a low dose (0.3 mg kg^−1^) of SUM-HPBCD observed in the von Frey test, similar to sumatriptan succinate and more effective than in its pure form, is in line with the results of the permeability experiments which showed superior permeability of SUM-HPBCD over the SUM-BCD form. Taken together, our present chemical and pharmacological study justifies the suggestion that the greater bioavailability of sumatriptan, in particular SUM-HPBCD, results from its inclusion into cyclodextrin structures.
